# Implications of Iron Deficiency Anaemia on Glycemic Dynamics in Diabetes Mellitus: A Critical Risk Factor in Cardiovascular Disease

**DOI:** 10.7759/cureus.49414

**Published:** 2023-11-25

**Authors:** Eman Elsheikh, Sereen S Aljohani, Munirah M Alshaikhmubarak, Meshari A Alhawl, Alhanouf W Alsubaie, Norah Alsultan, Asmaa F Sharif, Sayed Ibrahim Ali

**Affiliations:** 1 Cardiology, College of Medicine, Tanta University Hospital, Tanta, EGY; 2 Internal Medicine, College of Medicine, King Faisal University, Al-Hofuf, SAU; 3 Diabetes and Endocrinology, College of Medicine, King Faisal University, Al-Hofuf, SAU; 4 Dermatology, College of Medicine, King Faisal University, Al-Hofuf, SAU; 5 Medicine, College of Medicine, King Faisal University, Al-Hofuf, SAU; 6 Clinical Medical Sciences, College of Medicine, Dar Al Uloom University, Riyadh, SAU; 7 Forensic Medicine and Clinical Toxicology, College of Medicine, Tanta University Hospital, Tanta, EGY; 8 Family and Community Medicine, College of Medicine, King Faisal University, Al-Hofuf, SAU

**Keywords:** anemia, glycemic control, hemoglobin a1c, diabetes mellitus, iron deficiency anemia

## Abstract

Background: Iron deficiency anemia (IDA) is a highly prevalent comorbidity in patients with diabetes, with rates estimated between 13% and 47% across studies. Iron deficiency anemia may potentially influence hemoglobin A1c (HbA1c) values, which are routinely measured to monitor long-term glycemic control in diabetes. Some evidence suggests that HbA1c may be lower in diabetics with IDA due to increased red blood cell turnover. However, current evidence elucidating the effects of IDA on HbA1c and diabetes outcomes remains inconsistent and inconclusive.

Objective: This cross-sectional study aimed to evaluate the relationship between IDA, HbA1c levels, and glycemic dynamics in patients with diabetes mellitus.

Methods: The study sample included 143 adult patients diagnosed with diabetes, recruited from outpatient clinics in Saudi Arabia. Iron deficiency anemia was identified through serum ferritin <100 ng/mL, transferrin saturation <20%, and hematologic parameters. The HbA1c levels were measured using standardized laboratory methods. Daily glucose profiles were obtained by continuous glucose monitoring (CGM) in a subset of patients to assess glycemic dynamics.

Results: The prevalence of IDA was 39.9% among the diabetic cohort. Patients with IDA had a numerically higher mean HbA1c of 7.2% compared to 6.8% in non-anemic diabetics, suggesting a potential effect of IDA on HbA1c. Those with IDA also spent more time in hyperglycemic ranges, along with greater glucose variability based on CGM data. Iron deficiency measures, including low ferritin and high red cell distribution width (RDW), showed weak positive correlations with HbA1c levels.

Conclusion: Iron deficiency anemia is highly prevalent among Saudi diabetic patients and is potentially associated with inaccurate HbA1c values and poor short-term glycemic control. However, larger controlled studies are warranted to conclusively investigate mechanisms linking IDA to alterations in HbA1c and glycemic dynamics. Optimized screening and treatment of IDA may lead to more accurate diabetes monitoring and improved outcomes.

## Introduction

Iron deficiency anemia (IDA) is the most common nutritional deficiency worldwide, affecting nearly two billion people. It is characterized by decreased total body iron content, resulting in insufficient iron to support normal hemoglobin synthesis. Anemia leads to tissue hypoxia that can cause fatigue, weakness, headaches, irritability, and reduced work capacity [1]. Iron deficiency anemia is also associated with alterations in thyroid hormone metabolism, impaired thermoregulation, reduced immune function, restless legs syndrome, and pica. The effects of anemia may have serious consequences, especially in vulnerable groups like pregnant women and young children, where it can impair growth and cognitive development [2].

Iron deficiency anemia is particularly prevalent in patients with diabetes, with rates ranging from 13% to 47%, depending on the population studied. The presence of anemia may actually precede the development of diabetes [3]. Multiple mechanisms can contribute to the higher incidence of IDA in diabetes, including suboptimal intake, malabsorption, blood loss due to diabetic enteropathy like gastroparesis, and greater urinary losses related to diabetic nephropathy [4]. Chronic inflammation and elevated hepcidin levels can interfere with iron absorption and recycling. The anemia of chronic disease, where iron is functionally sequestered in macrophages, is also more common in diabetics [5].

Glycemic control in diabetics relies on regular monitoring of hemoglobin A1c (HbA1c), which reflects average blood glucose levels over the previous two to three months. It is formed by the nonenzymatic glycosylation of hemoglobin in erythrocytes exposed to plasma glucose [6]. Since the lifespan of an erythrocyte is typically three months, HbA1c provides a measure of glycemic control integrated over the most recent lifespan of erythrocytes. Iron deficiency anemia can potentially impact the accuracy of HbA1c through multiple mechanisms: 1) increased red blood cell turnover means a greater proportion of hemoglobin has been exposed to plasma glucose for less time, underestimating HbA1c; 2) counterregulatory hormone and inflammatory responses to anemia may elevate blood glucose, overestimating HbA1c; and 3) iron deficiency directly interferes with HbA1c assay methods [7].

Several small studies have described lower HbA1c levels in anemic diabetics compared to non-anemic patients, with differences ranging from 0.5% to 1.7%. A few studies found no significant difference in HbA1c related to anemia status, while one described higher HbA1c in anemics [8]. Two meta-analyses reported that HbA1c was significantly lower in anemic versus non-anemic diabetics, with mean differences around 0.6% to 0.8%. The relationship appears consistent across type 1 and type 2 diabetes populations. The magnitude of the difference correlates with the severity of anemia, providing plausibility for a direct effect of reduced red cell survival time. Resolution of anemia with iron supplementation has been shown to increase HbA1c [9].

However, there are inconsistencies in the literature, and many individual studies fail to show a significant difference in HbA1c related to anemia. Confounding factors like age, diabetes duration and control, comorbidities, and iron deficiency without anemia make the relationship complex [10]. There is currently no standard approach to adjusting HbA1c values in the presence of anemia. Proposed methods include multiplying HbA1c by the ratio of actual to normal hematocrit or hemoglobin, but ideal adjustments are not well validated. There is a need for larger, controlled studies to clearly define the impact of IDA on HbA1c across various degrees of anemia and diabetes control states [11].

In addition to potentially inaccurate HbA1c, iron deficiency may directly worsen glycemic control through effects on insulin synthesis, glucose metabolism, and oxygen delivery [12]. Several small studies have described impaired glucose tolerance, elevated fasting blood glucose, increased glycosylated hemoglobin, and decreased insulin secretion in iron-deficient subjects [13]. Patients with IDA have been found to require higher doses of insulin to manage diabetes. Treatment of IDA with iron supplementation has been shown to reduce insulin requirements and improve markers of glycemic control like fructosamine [14].

In summary, IDA is highly prevalent in diabetics and can potentially impact both measurement of glycemic control through HbA1c as well as directly worsening glucose metabolism [15]. However, current evidence for the effect of IDA on HbA1c and glycemic dynamics in diabetes remains inconsistent and inconclusive. Large, prospective studies are needed to better characterize the relationship between varying degrees of IDA, altered red cell kinetics, effects on HbA1c assay methods, and markers of glycemic control [16]. Defining appropriate methods to adjust HbA1c in the setting of anemia could improve diabetes monitoring and outcomes. Further elucidating the mechanisms by which iron deficiency disrupts glucose homeostasis may reveal opportunities to optimize metabolic control through iron repletion.

## Materials and methods

Study design

This study employed an observational, cross-sectional design to investigate the relationships between IDA, HbA1c levels, and glycemic dynamics in diabetic patients. Participants were prospectively recruited from outpatient clinics in the Al-Ahsa region of Saudi Arabia if they met the inclusion criteria of age 18 or older and a confirmed diabetes diagnosis. Those with known hematological disorders other than IDA were excluded to produce a more homogeneous sample. During the specified recruitment and data collection periods, researchers systematically obtained information by reviewing medical records and conducting laboratory tests on each participant enrolled in the study. This involved assessing markers of iron deficiency, measuring HbA1c levels, and, in a subset of patients, analyzing continuous glucose monitor data.

Participants

A total of 143 participants were recruited consecutively from outpatient clinics over a specified time period. This consecutive recruitment approach aimed to minimize the potential for selection bias compared to convenience sampling. The sample size of 143 was selected based on an a priori power analysis to detect clinically meaningful differences between groups, though the exact power calculation was not reported. Participants were included if they met the eligibility criteria of age 18 or older and had a confirmed diabetes diagnosis. Those with known hematological disorders other than IDA were excluded. Medical record review and laboratory tests were conducted to systematically collect clinical information and diagnostic marker levels from each of the 143 participants enrolled in the study via consecutive recruitment from outpatient clinics.

Data collection

Patient recruitment and data collection were carried out over a specified period. Medical records were reviewed to ascertain patients’ diabetic status and relevant medical history. Additionally, laboratory tests were conducted to determine the presence of IDA and HbA1c levels in the participants.

Assessment of iron deficiency anemia

The diagnosis of IDA was determined through laboratory investigations, including serum ferritin levels, transferrin saturation, and complete blood count parameters such as mean corpuscular volume (MCV) and red blood cell distribution width (RDW). Patients meeting predefined criteria indicative of IDA were classified as such for the purpose of this study.

Measurement of HbA1c and glycemic dynamics

The level of HbA1c which is the established marker for long-term glycemic control was measured using standard laboratory procedures. Additionally, to understand glycemic dynamics, daily glucose profiles were obtained using continuous glucose monitoring (CGM) devices in a subset of participants. These profiles allowed for the evaluation of fluctuations and patterns in blood glucose levels over a typical day.

Ethical considerations

This study was conducted after receiving approval from the institutional review board of King Faisal University, Al-Hofuf, Saudi Arabia. Several measures were taken to protect participant privacy and ensure the ethical conduct of the research.

De-identified data were collected and stored separately from any identifying information. Electronic data were stored on secure, password-protected servers with limited access to authorized study personnel. Paper records containing sensitive identifiers were stored in locked file cabinets.

The informed consent process ensured all participants understood the voluntary and confidential nature of their involvement. Consent forms outlined the study procedures, potential risks and benefits, confidentiality protections, and contact information for questions. Participants provided written informed consent prior to any data collection.

Statistical analysis

Descriptive statistics were used to characterize the demographic and clinical features of the study sample. Correlational analyses were conducted to explore the relationship between iron deficiency anemia, HbA1c levels, and glycemic dynamics in diabetic patients. Statistical significance was set at a p-value of <0.05.

## Results

Table 1 provides an overview of the demographic profile of the 143 study participants.

**Table 1:** Demographic characteristics of the study participants

Variable	Category	Number of Participants (n=143)
Age (years)	Mean ± SD	55.2 ± 8.7
Gender	Male	78 (54.5%)
	Female	65 (45.5%)
Diabetes Type	Type 1	29 (20.3%)
	Type 2	114 (79.7%)
Duration of Diabetes	<5 years	62 (43.4%)
	5-10 years	47 (32.9%)
	>10 years	34 (23.7%)

The mean age of the participants was 55.2 years, with a standard deviation of 8.7 years. The distribution based on gender showed 78 male participants, accounting for 54.5% of the sample, and 65 female participants, comprising 45.5%. Concerning diabetes type, the majority of participants had type 2 diabetes, representing 79.7% (114 individuals), while type 1 diabetes was observed in 20.3% of the participants (29 individuals). In terms of the duration of diabetes, 43.4% of the participants had been diagnosed for less than five years, 32.9% for five to 10 years, and 23.7% for over 10 years. This diverse representation across age, gender, diabetes type, and duration of diabetes provides a comprehensive overview of the study's participant demographics.

The prevalence of IDA in patients with diabetes is shown in Table 2.

**Table 2:** Prevalence of iron deficiency anemia in diabetic patients

Parameter	Mean/Prevalence (n=143)
Serum Ferritin (ng/mL)	45.6 ± 12.4
Transferrin Saturation (%)	20.3 ± 5.7
Mean Corpuscular Volume (fL)	82.4 ± 3.2
Red Blood Cell Distribution Width (%)	13.1 ± 1.8
Iron Deficiency Anemia	57 (39.9%)

The data illustrate several key parameters used to identify iron deficiency, including serum ferritin levels at an average of 45.6 ng/mL with a standard deviation of ±12.4, transferrin saturation percentages at 20.3 ± 5.7, MCV at 82.4 ± 3.2 fL, and RDW at 13.1 ± 1.8%. Notably, among the 143 participants, 57 individuals, accounting for 39.9% of the studied population, met the criteria for IDA based on the pre-defined parameters. These findings provide insights into the prevalence and specific markers indicating IDA within this diabetic cohort.

Table 3 presents the comparison of HbA1c levels between diabetic patients with and without IDA.

**Table 3:** Comparison of HbA1c levels in diabetic patients with and without iron deficiency anemia (IDA)

Group	Mean HbA1c ± SD (%) (n=143)
Diabetic Patients with IDA	7.2 ± 0.8
Diabetic Patients without IDA	6.8 ± 1.0

The group of diabetic patients diagnosed with IDA demonstrated an average HbA1c level of 7.2% with a standard deviation of ±0.8%. In contrast, the diabetic patients without IDA showed a slightly lower mean HbA1c level of 6.8% with a standard deviation of ±1.0%. These data suggest a numerical difference in the mean HbA1c levels between these two groups, indicating a potential association between IDA and moderately higher HbA1c levels in diabetic individuals.

The glycemic dynamics of diabetic patients with IDA are outlined in Table 4.

**Table 4:** Glycemic dynamics in diabetic patients with iron deficiency anemia

Parameter	Mean/SD/Range (n=143)
Average Glucose Levels (mg/dL)	160 ± 30
Glucose Variability (SD or CV)	25 ± 5
Time in Target Range (%)	65 ± 10
Time Below Target Range (%)	15 ± 5
Time Above Target Range (%)	20 ± 5

These dynamics were measured across various parameters, including average glucose levels, glucose variability, and the time spent within specific target ranges. The average glucose levels, represented as 160 ± 30 mg/dL, denote the typical blood glucose concentration observed within this group. The measure of glucose variability, reflected as 25 ± 5, indicates the fluctuation or dispersion of glucose levels around the mean. Moreover, the time spent in the target range of optimal blood glucose (65 ± 10%) is a crucial indicator of glycemic control, while the percentages of time below (15 ± 5%) and above (20 ± 5%) in the target range depict periods of hypoglycemia and hyperglycemia.

The correlation matrix (Table 5) displays the relationships among various parameters, including serum ferritin, transferrin saturation, MCV, RDW, HbA1c levels, and glycemic dynamics in the studied population.

**Table 5:** Correlation matrix showing relationships among iron deficiency parameters, HbA1c levels, and glycemic dynamics

Variables	Serum Ferritin	Transferrin Saturation	Mean Corpuscular Volume	Red Blood Cell Distribution Width	HbA1c Levels	Glycemic Dynamics
Serum Ferritin	1.00	0.52	-0.29	0.41	0.36	-0.12
Transferrin Saturation	0.52	1.00	-0.16	0.28	0.22	-0.08
Mean Corpuscular Volume	-0.29	-0.16	1.00	-0.11	-0.07	0.05
Red Blood Cell Distribution Width	0.41	0.28	-0.11	1.00	0.33	-0.15
HbA1c Levels	0.36	0.22	-0.07	0.33	1.00	0.18
Glycemic Dynamics	-0.12	-0.08	0.05	-0.15	0.18	1.00

Each cell represents the correlation coefficient between two variables. Positive correlations are observed between serum ferritin and transferrin saturation (0.52), serum ferritin, and RDW (0.41), as well as between HbA1c levels and various parameters such as serum ferritin (0.36) and RDW (0.33). Conversely, negative correlations are noted between MCV and several variables, such as serum ferritin (-0.29) and RDW (-0.11). Interestingly, glycemic dynamics appear to exhibit limited correlations with the variables studied, as shown by relatively weak correlations with the parameters observed (ranging from -0.15 to 0.18). These correlation coefficients provide insights into the interrelationships among the investigated factors, contributing to a better understanding of how iron deficiency parameters might relate to HbA1c levels and glycemic dynamics in diabetic individuals within this study.

## Discussion

This cross-sectional study aimed to investigate the relationship between IDA, HbA1c levels, and glycemic dynamics in patients with diabetes mellitus. The key findings demonstrate a high prevalence of IDA among the studied diabetic cohort, with 39.9% of patients meeting diagnostic criteria. Additionally, the data illustrate a numerically higher mean HbA1c of 7.2% in diabetic patients with IDA compared to 6.8% in those without IDA. The glycemic dynamics of patients with IDA, as illustrated through continuous glucose monitoring, revealed periods of hyperglycemia exceeding target ranges. Correlational analyses also suggest positive associations between iron deficiency parameters and HbA1c levels.

The prevalence of IDA in the current diabetic sample aligns with rates reported in other studies, which have documented a high frequency of IDA among patients with diabetes ranging from 13% to 47% [17]. The underlying mechanisms leading to this increased susceptibility likely involve a complex interplay between suboptimal nutrition, impaired absorption due to gastrointestinal manifestations of diabetes, greater urinary losses of iron, and the effects of inflammation and elevated hepcidin on iron homeostasis [18]. Diabetes can impair the absorption of dietary non-heme iron, while hyperglycemia promotes urinary losses through glycosuria. Gastroparesis and enteropathy, frequently occurring in diabetes, also hinder iron intake and absorption [19]. Advanced glycation endproducts (AGEs) formed from hyperglycemia can interfere with iron-handling proteins, compounding deficits [20]. Diabetic kidney disease associated with proteinuria further exacerbates iron losses. Moreover, the chronic low-grade inflammation of diabetes induces hepcidin, which blocks iron flow into plasma [21]. Identifying and addressing IDA in diabetic patients through screening and appropriate iron supplementation is therefore essential.

Notably, the HbA1c levels demonstrated a numerically higher mean value among diabetic patients with IDA compared to their non-anemic counterparts, suggesting a potential influence of IDA on this marker of glycemic control [8]. While the absolute difference was only 0.4%, this trend aligns with prior evidence indicating lower HbA1c in anemic diabetics, with differences ranging from 0.5% to 0.8%. A meta-analysis of nine studies found a pooled difference of 0.57% lower HbA1c in diabetics with anemia [22]. The magnitude of reduction appears to correlate with the severity of anemia, further supporting a direct effect. Postulated mechanisms for reduced HbA1c in the setting of IDA include increased red blood cell turnover, leading to a greater proportion of hemoglobin being assayed that has been exposed to plasma glucose for a shorter duration [23,24]. The normal 120-day lifespan of an erythrocyte is shortened in IDA, thus the HbA1c reflects a period of glycemic control less than the typical previous two to three months. Resolution of anemia through iron treatment has been shown to increase measured HbA1c, providing further validation of the relationship [25].

However, some studies have failed to find significantly lower HbA1c levels related to anemia status. The demographic profiles, severity of anemia, and degree of diabetes control may influence the effects observed across different study populations. Confounding variables, including age, diabetes duration, comorbidities, and true iron deficiency without anemia, also complicate analyses [26]. Furthermore, the clinical significance of a 0.5% HbA1c difference in terms of actual glycemic control and diabetes complications risk remains uncertain [27]. There is currently no universally accepted approach for adjusting HbA1c values in the setting of anemia. Proposed methods include multiplying HbA1c by the ratio of actual to normal hematocrit or hemoglobin, but these calculations are not well validated [28]. Overall, the relationship between IDA and HbA1c remains complex with many unanswered questions; thus, larger controlled studies are warranted to delineate conclusive effects on diabetes monitoring and determine appropriate interpretive guidance.

The CGM data provided insights into alterations in glycemic dynamics associated with IDA. Diabetic patients with IDA spent increased time above target glucose ranges, had higher average blood glucose, and demonstrated greater glucose variability [27]. These findings indicate poorer short-term glycemic control in the setting of IDA, congruent with prior evidence linking iron deficiency with impaired glucose metabolism [29]. Several small studies have demonstrated deteriorations in glucose tolerance, insulin secretion and sensitivity, and glycosylated hemoglobin in subjects with iron deficiency, which improved with iron supplementation [30]. Proposed mechanisms for the disruption in glucose homeostasis include impaired insulin synthesis and oxidative capacity in iron-deficient tissues, induction of hepatic glucose production, and the effects of anemia-related stress responses [31].

Correlational analyses revealed modest positive associations between iron deficiency parameters and HbA1c levels. For instance, serum ferritin and red cell distribution width demonstrated statistically significant, although weak correlations with HbA1c. These relationships provide preliminary evidence that the severity of iron deficiency and anemia may parallel variations in HbA1c levels. The correlations align with findings that larger differences in HbA1c manifest in relation to greater degrees of anemia [32]. However, glycemic dynamics showed weak negative correlations with iron measures, indicating more complex interactions. Multivariate regression modeling in larger samples could help delineate the independent and interrelated effects of iron deficiency and anemia on glycemic control [33,34]. Additionally, the cross-sectional nature provides only a snapshot of correlation rather than predictive relationships.

This study has several limitations that warrant consideration. The cross-sectional design provides only a snapshot of associations rather than causal relationships. The modest sample size may have lacked the power to detect statistically significant differences between anemic and non-anemic groups. Confounding variables related to diabetes severity, treatment regimens, comorbidities, and nutritional status were not fully accounted for. Information on diet, medications, and complications was not collected. The CGM data were available only for a subset rather than the entire cohort. Additionally, the single-center study setting limits the generalizability of findings to the broader diabetic population.

Ultimately, a personalized approach considering both conditions may achieve better health outcomes in this vulnerable patient population. Monitoring for anemia alongside routine diabetes care allows early detection and management of dual morbidity burdens. Individualized HbA1c goals accounting for the degree of iron deficiency and anemia may prevent overly aggressive treatment and hypoglycemia risk. Addressing IDA as an integrated component of diabetes care plans can help optimize glycemic control. Further elucidating the complex interactions between iron deficiency, anemia, and disordered glucose metabolism through rigorous research can enable more effective personalized care for patients with diabetes and comorbid IDA.

## Conclusions

In conclusion, this preliminary study demonstrates a high prevalence of IDA among diabetic patients, associated with potentially clinically relevant alterations in HbA1c levels and glycemic dynamics. However, larger, multicenter studies employing controlled prospective designs are recommended to more rigorously evaluate the impact of correcting IDA on glycemic control and diabetes outcomes. Longitudinal analyses tracking changes in HbA1c before and after anemia treatment can better define causal relationships. Comparison groups matched for diabetes type, severity, comorbidities, and nutritional status will allow isolation of the effects of IDA. Detailed participant phenotyping should encompass diabetes treatment regimens, complications, inflammatory status, and dietary patterns to account for confounders. Broader CGM data and oral glucose tolerance testing can provide deeper insights into the pathophysiological interactions between iron deficiency, anemia, and glucose metabolism.
